# Levofloxacin HCl-Incorporated Zein-Based Solvent Removal Phase Inversion In Situ Forming Gel for Periodontitis Treatment

**DOI:** 10.3390/pharmaceutics15041199

**Published:** 2023-04-10

**Authors:** Setthapong Senarat, Catleya Rojviriya, Napaphol Puyathorn, Nutdanai Lertsuphotvanit, Thawatchai Phaechamud

**Affiliations:** 1 Programme of Pharmaceutical Engineering, Faculty of Pharmacy, Silpakorn University, Nakhon Pathom 73000, Thailand; 2 Synchrotron Light Research Institute, Mueang District, Nakhon Ratchasima 30000, Thailand; 3 Program of Pharmaceutical Technology, Department of Pharmaceutical Technology, Faculty of Pharmacy, Silpakorn University, Nakhon Pathom 73000, Thailand; 4 Department of Industrial Pharmacy, Faculty of Pharmacy, Silpakorn University, Nakhon Pathom 73000, Thailand; 5 Natural Bioactive and Material for Health Promotion and Drug Delivery System Group (NBM), Faculty of Pharmacy, Silpakorn University, Nakhon Pathom 73000, Thailand

**Keywords:** zein, in situ forming gel, levofloxacin, solvent removal, periodontitis

## Abstract

Zein is composed of nonpolar amino acids and is a water-insoluble protein used as the matrix-forming agent of localized in situ forming gel (ISG). Therefore, this study prepared solvent removal phase inversion zein-based ISG formulations to load levofloxacin HCl (Lv) for periodontitis treatment using dimethyl sulfoxide (DMSO) and glycerol formal (GF) as the solvents. Their physicochemical properties were determined, including viscosity, injectability, gel formation, and drug release. The topography of dried remnants after drug release was revealed using a scanning electron microscope and X-ray computed microtomography (μCT) to investigate their 3D structure and % porosity. The antimicrobial activities were tested against *Staphylococcus aureus* (ATCC 6538), *Escherichia coli* ATCC 8739, *Candida albicans* ATCC 10231, and *Porphyromonas gingivalis* ATCC 33277 with agar cup diffusion. Increasing zein concentration or using GF as the solvent notably enhanced the apparent viscosity and injection force of the zein ISG. However, its gel formation slowed due to the dense zein matrix barrier’s solvent exchange: the higher loaded zein or utilization of GF as an ISG solvent prolonged Lv release. The SEM and μCT images revealed the scaffold of dried ISG in that their % porosity corresponded with their phase transformation and drug release behavior. In addition, the sustainability of drug diffusion promoted a smaller antimicrobial inhibition clear zone. Drug release from all formulations was attained with minimum inhibitory concentrations against pathogen microbes and exhibited a controlled release over 7 days. Lv-loaded 20% zein ISG using GF as a solvent exhibited appropriate viscosity, Newtonian flow, acceptable gel formation and injectability, and prolonged Lv release over 7 days with efficient antimicrobial activities against various test microbes; thus, it is the potential ISG formulation for periodontitis treatment. Consequently, the Lv-loaded solvent removal zein-based ISGs proposed in this investigation offer promising potential as an efficacious drug delivery system for periodontitis treatment by local injection.

## 1. Introduction

Zein is a vegetal protein extracted from maize that has been promised as a suitable biomaterial for biomedical and pharmaceutical applications owing to its biodegradability and biocompatibility. Zein comprises nonpolar amino acids, including leucine, proline, alanine, and phenylalanine; thus, this protein is water-insoluble [1,2]. Moreover, this material has strongly defined hydrophobic and hydrophilic regions on its structural surface and can self-assemble into a wide range of meso-structures. The hydration of zein requires a rather high temperature to function as a viscoelastic polymer because of its high glass transition temperature [3,4]. It is composed of a three-dimensional network aligned in two ways: cylindrical and ribbon-like models in which the hydrophobic helices are arranged edgewise, and the hydrophilic glutamine bridges connect the top and bottom surfaces of the repeat units [5]. Zein stands out from other proteins because it has only traces of lysine, tryptophan, arginine, and histidine. This amino acid composition is the reason for its unique solubility, mainly restricted to acetone, acetic acid, aqueous alcohols, and aqueous alkaline solutions [5,6]. The US FDA approved zein as a Generally Recognized As Safe (GRAS) material for utilization focused mostly on its industrial and consumer applications such as plastics, inks, molded products, gum, printing film, paper, and food coatings [2,6]. Extensive research has revealed that zein acts as an efficient excipient to control the delivery of essential oils, dyes, antimicrobial agents, anticancer agents, macrophage targeting, oral drug delivery with stomach protection, and gene delivery as micro/nanoparticles [7,8,9,10]. In addition, zein exhibits good degradation properties and may be scrutinized as a structural material in implants [11]. Zein, being a protein, is degraded by numerous enzymes, including pepsin and pancreatic enzymes, depending on the application site. Zein is digested in the oral cavity by protein-degrading enzymes such as collagenase and trypsin-like enzymes found in saliva and gingival crevicular fluid [12,13].

Moreover, zein has been reported to be a favorable biomaterial with good biocompatibility for tissue engineering [14]. Practically, it has been fabricated into a strong, glossy coating with antibacterial activity and is commonly used in food and pharmaceutical industries [10,15]. Furthermore, zein exhibits mucoadhesive properties and an ability to sustain the drug in the gastrointestinal environment, making it suitable for mucosal drug administration [16]. In addition, self-assembled zein organogel has been mentioned for developing 3D-printed drug delivery systems or scaffolds for tissue engineering [17]. The controlled drug delivery of small molecules, fabrication of bioactive membranes, and 3D assembly of scaffolds to regenerate tissue with zein via micro- or nano-structure templates are currently undergoing extensive investigation in the biomedical world [18].

Dual-drug-loaded solvent-induced phase inversion-induced in situ forming implant was fabricated to deliver pitavastatin (osteogenic drug) and tedizolid (antibiotic) using zein as the implant matrix for bone healing [19]. Gao et al. [20] prepared a zein-based in situ gelling system containing pingyangmycin to treat venous malformations. The interstitial chemotherapy using a doxorubicin-loaded zein-based in situ gel system regulates drug release over time after intratumoral injection [21]. The solvent casting/particulate leaching procedure created a zein porous scaffold to promote the growth of periodontal ligament cells because it was biocompatible and well interconnected [22]. The current research’s conceptual framework is focused on applying zein as a matrix-forming agent for the solvent removal phase inversion-based in situ forming gel (ISG) system loaded with an antimicrobial agent for periodontitis treatment. This developed drug delivery system has not been reported previously. Zein is an interesting protein to apply as a matrix-forming agent of this type of ISG for crevicular pocket targeting. It is safe to use over long periods and for its apparent water insolubility but miscibility in an organic solvent.

Restricted access to periodontitis treatment typically occurs to reach the periodontopathogens, especially Gram-negative anaerobic bacteria such as *Porphyromonas gingivalis* and *Actinobacillus actinomycetemcomitans*, in the deep periodontal pocket in which they are naturally obtrusive. Antibacterial therapy has been used as an adjuvant for conventional mechanical grafting [23,24]. To conquer the cons of systemic antimicrobial use, local site administration of antimicrobial delivery has attained prodigious attention intending to deliver and maintain the adequate therapeutic dose of the drug for a sustained period while minimizing its side effects [25]. Solvent removal phase inversion-induced ISG is gaining widespread attention as an engaging injectable dosage form for periodontitis treatment due to its simplicity of administration and less complicated preparation in accordance with the presence of the matrix-forming agent to prolong drug release [26,27]. The matrix-forming agents should be water-insoluble, biocompatible, biodegradable, and miscible with the solvent and drugs [28]. This drug delivery system consists of a drug-loaded polymer solution that can solidify via solvent removal phase inversion and turn into a drug-entrapped gel-like or polymer matrix depot in the periodontal pocket via injection by exposure to the aqueous periodontal fluids [29,30].

**Figure 1:**
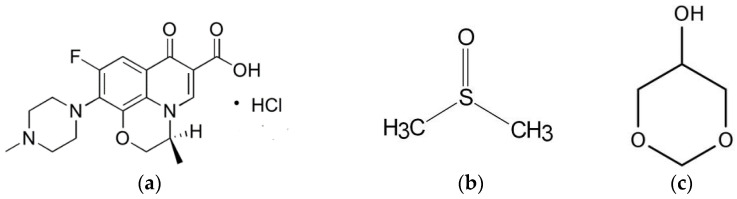
Chemical structure of levofloxacin HCL (Lv) (**a**), dimethyl sulfoxide (DMSO) (**b**), and glycerol formal (GF) (**c**).

Levofloxacin HCL (Lv) (Figure 1a) is a broad-spectrum, third-generation fluoroquinolone antibiotic that inhibits bacterial DNA gyrase and topoisomerase IV, enzymes required for DNA replication [31]. Lv is active against a wide range of aerobic Gram-positive and Gram-negative organisms and demonstrates moderate activity against anaerobes [32]. Unique animal bite wound isolates such as *Porphyromonas macaccae* and *P. gingivalis,* and *Prevotella heparinolytica* are usually susceptible to 0.25 and 0.5 mg/mL of levofloxacin, respectively [33]. Levofloxacin administered with a combination drug in situ gel-forming nasal delivery system on repairing nasal mucosa damage has been reported to prevent stenosis of the new ostia, significantly reduce chronic inflammation, and actively support wound healing [34]. Chitosan and poloxamer in situ gel containing levofloxacin and metronidazole sustained drug release for up to 48 h [35]. In addition, the medicated in situ forming gel showed better clinical outcomes than scaling and root planing alone [36]. From a preliminary study, this drug could dissolve in dimethyl sulfoxide (DMSO) (Figure 1b) and glycerol formal (GF) (Figure 1c). Thus, levofloxacin HCl is interesting as an antimicrobial drug of solvent removal phase inversion ISG for treating periodontitis due to its broad spectrum and good activity against selective anaerobic bacteria.

This study aimed to develop the solvent removal phase inversion ISG formulation using zein as the matrix-forming agent for periodontitis treatment. To achieve formulation development, understanding the matrix-forming behavior of these agents was investigated. Thus, the influence of matrix concentrations was studied and discussed in terms of physicochemical properties, matrix-forming behavior, and antimicrobial activities. In addition, the physicochemical characteristics of prepared Lv-loaded ISG formulations were investigated via apparent viscosity, rheology behavior, injectability, matrix formation behavior, matrix morphology, drug release, and antimicrobial activities.

## 2. Materials and Methods

### 2.1. Materials

Zein (lot. no. 9010-66-6) was purchased from Qingdao Sigma Chemical Co., Qingdao, China. Levofloxacin HCl was kindly supported by Siam Pharmaceutical Co., Bangkok, Thailand, and was used as an antimicrobial drug. DMSO (≥99.9%, Lot No. 1862992, Fisher Chemical, Horsham and Loughborough, UK) and GF (≥98.0%, Lot # BCCD7726, Sigma-Aldrich, Overijse, Belgium) were used as the solvents. Agarose (Lot No. H7014714, Vivantis, Selangor Darul Ehsan, Malaysia) was used to analyze the behavior of gel formation. Sheep blood agar (Ministry of Public Health, Nonthaburi, Thailand), tryptic soy agar, and tryptic soy broth (Difco^™^, Detroit, MI, USA) were used as media for antimicrobial investigations. Sabouraud dextrose agar (SDA) and Sabouraud dextrose broth (SDB) (Difco, Detroit, MI, USA) were employed for the antifungal test. Potassium dihydrogen orthophosphate (lot no. E23W60, Ajax Finechem, New South Wales, Australia) and sodium hydroxide (lot no. AF310204, Ajax Finechem, New South Wales, Australia) were used as components in the phosphate-buffered saline (PBS). *S. aureus* ATCC 6538, *E. coli* ATCC 8739, *C. albicans* ATCC 10,231 (Department of Medical Sciences, Ministry of Public Health, Nonthaburi, Thailand), and *P. gingivalis* ATCC 33,277 (Microbiologics Inc., St Cloud, MN, USA) were procured from Thai Can Biotech Co., Ltd., Bangkok, Thailand) and used as test microbes.

### 2.2. Preparation of In Situ Forming Gel

ISGs comprising zein at various concentrations of 20–25% *w*/*w* dissolved in DMSO and GF were produced by stirring them continuously. Next, 1% *w*/*w* Lv was added to the prepared zein solutions. The components of all formulations are shown in Table 1.

### 2.3. Rheology and Viscosity Characterization

The viscosity values were measured every 15 sec at different equilibration time shear rates using a viscometer cone-plate RM 100 CP2000 plus (Lamy Rheology Instruments Company, Champagne-au-Mont-d’Or, France). Rheological data was also recorded with this instrument. The shear stress of the ISG solutions was undertaken with several shear rates at room temperature (*n* = 3).

### 2.4. Injectability 

An injectability test was undertaken to indicate the ease of injection. The compression mode of a texture analyzer (TA.XT plus, Stable Micro Systems, Godalming, UK) was applied to determine an expelling force of formulation from a 1 mL plastic syringe coupled with an 18-gauge stainless needle. The upper probe of this instrument pressed the syringe plunger at a constant force of 0.1 N and speed of 1.0 mm/s until the syringe’s barrel base was reached. The maximum expelling force measured the force required for the sample injection fluid from a syringe via the needle, and the expulsion energy was determined from force-displacement profiles. The experiments were conducted in triplicate.

### 2.5. Gel Formation Study

The gel formation was detected macroscopically by injecting the prepared formulations through a 1-mL plastic syringe through an 18-gauge needle into PBS (pH 6.8) in a glass test tube to investigate matrix formation at the time of formulation by photographing it at various times (0, 1, 5, 10, 20 and 30 min). Moreover, the 0.6% agarose solution prepared by dissolving agarose in PBS (pH 6.8) was poured onto petri dishes (agarose gel height of 1 cm). After gel setting, it was cut with a cylinder cup into a 300 μL hole (diameter of 7 mm) at the center of the petri dish. This agarose gel represented an artificial periodontal pocket in humans. A 150 μL aliquot of the ISG formulations was added to the fabricated cylindrical agarose well. After being in contact with the agarose gel, the solvent exchange formed a cloudy matrix during phase separation. The zein matrix formation was photographed under a stereo microscope (SZX10, Olympus Corp., Tokyo, Japan) at 1, 5, 10, 20, and 30 min.

### 2.6. Drug Content and In Vitro Drug Release Studies

The amount of Lv content in prepared samples was analyzed from the standard curve using UV- spectrophotometer (Cary 60 UV-Vis, Model G6860A, Agilent, Petaling Jaya, Selangor, Malaysia) at a wavelength of 287 nm. (*n* = 6). The drug release from developed ISGs was tested and compared with the control formulation (1% *w*/*w* Lv dissolved in DMASO and GF). A cylindrical porcelain cup (diameter of 1 cm and height of 1.2 cm) containing 0.4 g of the ISG was cautiously placed in 50 mL of PBS (pH 6.8) at 37 °C, and the speed of the rotation shaker was maintained at 50 rpm to simulate the drug release behavior from the periodontal pocket. The release medium was sampled (5 mL) and replaced with 5 mL fresh PBS. The release amount of Lv was determined with a UV-visible spectrophotometer (Cary 60 UV-Vis, Model G6860A, Agilent, Petaling Jaya, Selangor, Malaysia) at a wavelength of 287 nm. and calculated into % cumulative drug release. The experiments were conducted in triplicate.

The drug release kinetic was undertaken by fitting the dissolution profiles with mathematical models, which included zero order, first order, Higuchi’s and Korsmeyer–Peppas. The DD-Solver software application, an add-in program for Microsoft Excel (Microsoft Corporation, Redmond, WA, USA), was employed for release profile fitting. The value of the release exponent (*n*-value) from the Korsmeyer–Peppas equation was used to indicate the drug release mechanism.

### 2.7. Scanning Electron Microscopy (SEM)

The morphology of the zein ISG system was investigated using SEM (TESCAN MIRA3, Brno-Kohoutovice, Czech Republic) at an accelerating voltage of 15 kV. After the drug release, the ISG remnant was washed with 200 mL of distilled water and freeze-dried using a freeze dryer (Triad^™^ Labconco, Kansas City, MO, USA). The dried ISG remnants were stored in a desiccator for one week and coated with gold prior to examination via the SEM technique and compared with the intact Lv powder.

### 2.8. X-ray Imaging and X-ray Tomographic Microscopy

After a drug release period of 7 days, the dried ISG remnants were prepared using a freeze dryer (Triad^™^ Labconco, Labconco Corporation, Kansas City, MO, USA). They were kept in a desiccator at room temperature for one week to avoid the melting and collapse of their structures, as mentioned in 4.7. These prepared dried ISG remnants were investigated with X-ray imaging and X-ray tomographic microscopy at the X-ray tomographic microscopy (XTM) beamline, Synchrotron Light Research Institute (SLRI), Nakhon Ratchasima, Thailand. The X-ray beam was generated from a 2.2-T multipole wiggler at the 1.2-GeV Siam Photon Source facility, which was operated at a current of 150 mA. The synchrotron radiation X-ray tomographic microscopy (SRXTM) tests were conducted using a filtered polychromatic X-ray beam, at the mean energy of 11.5 kV, with a source-to-sample distance of 34 m. All X-ray projections of the samples were attained from the detection system, which was equipped with a 200 µm thick scintillator (YAG:Ce, Crytur, Turnov, Czech Republic), lens-coupled X-ray microscope, and the sCMOs camera (PCO edge 5.5, 2560 × 2160 pixel, 16 bits) (Optique Peter, Lentilly, France). The tomographic scans were acquired at an isotropic voxel size of 3.61 µm. A tomographic volume of each sample was reconstructed from enlarged composite projections acquired from two scans. The initial scan was run over 180°. Then, the other 180° scan was performed on vertical axis of rotation that shifted horizontally and parallel to the camera. Then, the X-ray projections were normalized using a flat-field correction and reconstructed based on filtered back projection algorithm using the Octopus reconstruction software (TESCAN, Gent, Belgium). The 3D presentation of tomographic volumes of the composite films was determined using Drishti software (National Computational Infrastructure’s VizLab, Canberra, Australia). The segmentation analysis of porosity in 3D was determined by using Octopus Analysis (TESCAN, Gent, Belgium).

### 2.9. Antimicrobial Activities

The standard microbes in this experiment included *S. aureus* ATCC 6538, *E. coli* ATCC 8739, *C. albicans* ATCC 10231, and *P. gingivalis* ATCC 33277. The antimicrobial activities of the solvents, drug-free preparations, and Lv-loaded zein-based ISG formulations were undertaken using the agar diffusion assay (cylinder plate method). This technique relied on the diffusion of the test sample from a stainless-steel cylinder cup (with a diameter of 6 mm and a height of 10 mm) through agar media inoculated with the test microbes. The microbe inocula close to the 0.5 McFarland turbidity standard were spread on TSA and SDA, respectively. A 100 µL aliquot of each test sample was dropped into the cylinder cap, which was placed on the surface of an inoculated medium plate. For *P. gingivalis,* the bacterial inocula with turbidity close to the 0.5 McFarland turbidity standard were spread on sheep blood agar. The test was undertaken in an anaerobic incubator (Forma Anaerobic System, Thermo Scientific, Cincinnati, Ohio, USA). After incubating at 37 °C for 18 h, the diameter of the inhibition zone against these microbes was measured using a ruler (*n* = 3).

### 2.10. Statistical Analysis

All data were examined using the one-way analysis of variance (ANOVA) followed by the Tukey test. The value of *p* < 0.05 was considered statistically significant. The analysis was conducted using SPSS for Windows (version 11.5).

## 3. Results and Discussion

### 3.1. Viscosity and Rheological Behavior

**Figure 2:**
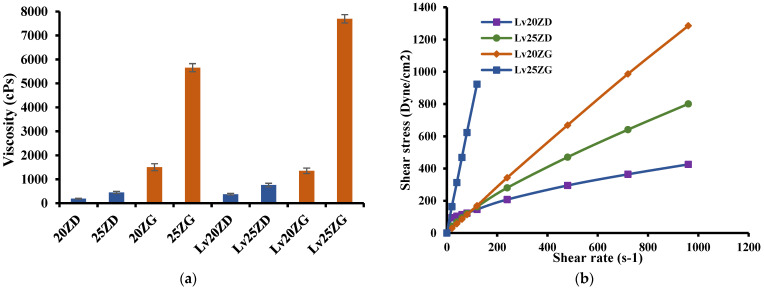
Viscosity (**a**) and relationship between shear stress and shear rates (**b**) of zein-loaded ISG formulations at 25 °C. The data is represented in triplicate.

The effect of zein concentrations on the apparent viscosity of ISG formulations is presented in Figure 2a. Increasing the zein concentration enhanced the apparent viscosity of the ISG formulations. The viscosities of the ISG formulations for Lv20ZD, Lv25ZD, Lv20ZG, and Lv25ZG were 376.37 ± 1.90, 759.09 ± 2.32, 1353.85 ± 14.37, and 7694 ± 6.08 cP, respectively. Moreover, using DMSO as a solvent resulted in lower viscosity due to the solvent being less viscous and zein more freely dissolving than in GF. Ordinally, a good solvent for dissolving the substance could diminish the viscosity of the mixture because the domination of substance–solvent interaction is over substance–substance interaction [37,38,39,40]. The nature of dissolved zein-promoted formulations was more viscous from its large protein structure in the solution [18]. Though virtually insoluble in water, zein not only presents as a globular protein in nonaqueous solutions but exhibits conformational characteristics of more conventionally behaving globular proteins [41]. The intrinsic viscosity of bleached shellac in NMP was higher than that in DMSO and PYR, respectively; thus, NMP exhibited as a good solvent for dissolving shellac [37,38]. Polymers present a different chain configuration in various dispersing fluids because of their different affinities with solvents [38]. The polymer/solvent interaction is normally outstanding for diluted polymeric solutions; viscosity increases with enhancing solvent power. On the other hand, with a higher polymer concentration, poor solvents have a higher viscosity than good solvents [39]. However, the viscosity of zein ISG systems increased when the drug was incorporated owing to less solvent in the system [40,41,42,43], resulting in the incremental viscosity of these systems. Polymers such as ethyl cellulose, bleach shellac, Eudragit RS, polylactide (PLA), and poly lactide-co-glycolide (PLGA) have been reported previously as the matrix-forming agents in ISG systems; however, these materials typically promote high viscous ISGs and poor injectability is evident [29,43]. For example, the viscosity of 25–35% (*w*/*w*) PLA and PLGA-based ISGs is approximately 44,200–204,400 cPs [43]. Thus, the zein-based ISG showed less viscosity. Nevertheless, the viscosity also affects the diffusion of species in the solidifying matrix. High viscosity normally slows down water entry and drug diffusion in this respect. Thus, it lowers the initial burst drug release and delays polymer degradation.

The rheological properties of prepared ISG formulations are shown in Figure 2b. All formulations showed a linear relationship between shear stress and shear rate. The curve moving to a higher shear stress value was due to the more compact zein structure of the formulations. Thus, the prepared formulations exhibited a Newtonian flow behavior. These results were in good agreement with the previous report on the Newtonian flow of polymer solutions [44]. In addition, the greater the 3D network formation within the zein molecule, the more it provoked a remarkable enhancement in the viscosity of these formulations [41,42]. As they have Newtonian flow behaviors, physicians or dentists have considered them suitable for injection dosage forms since an injection through a needle is acceptable after applying force to a syringe plunger to expel the formulation through the stainless needle [45].

### 3.2. Injectability

By dosage form design, ISGs are administered by targeted injection into the human body; therefore, the formula should be in a fluid state and painlessly injected [46]. In addition, the use of high force of expulsion indicates lower injectability. The force and work from the injectability check of the prepared formulations are displayed in Table 2. Significantly, ZG25 and LvZG25 had to apply a higher force and energy for injection than the other formulations (*p*-value < 0.05), especially zein in the DMSO formulations. Furthermore, as the zein concentration increased from 20% *w*/*w* to 25% *w*/*w* in both the DMSO and the GF, the work required to expel the formulation through the needles also increased significantly (*p* < 0.05), which corresponded with their viscosity results [47]. This demonstrated that the type of solvent and concentration of zein notably affected the required force and energy for injection. However, the results showed that most ISG formulations had a lower injection force (<5 N). Although the ISG system prepared using GF as a solvent had a greater force of injectability than the other formulations, it was easily injected through the tested needle. Even though zein-based ISG systems showed high viscosity due to their polymeric property, their expulsion work was apparently less than that of bleached shellac, ethyl cellulose, and Eudragit RS ISG systems [29,40]. Practically, the entire expulsion of each solution was less than 50 N.mm, demonstrating their acceptability for the criteria of injection [48].

### 3.3. Gel Formation

**Figure 3:**
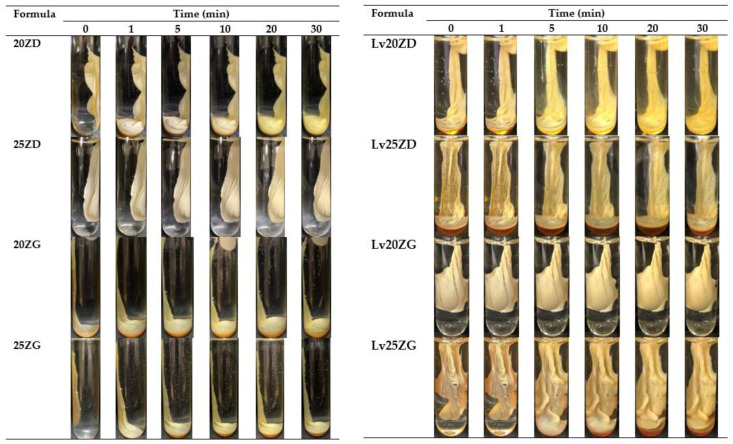
Gel formation of drug-free and Lv-loaded zein-based ISG formulations after injection into the phosphate buffer pH 6.8.

After injecting the formulation into PBS pH 6.8, the solvent exchange occurred. Therefore, the exterior part of all formulations immediately turned into opaque skin, and the gel transformed into a pale yellow opaque solid mass, as illustrated in Figure 3. Generally, the highly concentrated loading with polymer accelerated the phase separation rate of ISG [40]. When a formula with a greater concentration of zein (20% and 25%) contacted the PBS solution, it immediately transformed into a gel and sank to the bottom of the PBS. As the quantity of zein in the gel rose, it became cloudier and similar to the solid-like matrix. The solid-like opaque was also observed for poly (lactic-co-glycolic acid)-based ISG after exposure during the aqueous phase owing to its polymeric phase inversion [49].

Moreover, the solvent type (GF and DMSO) also affected the onset of the gel formation [50]. Zein in DMSO formulations transformed into a matrix faster than this protein in GF formulations. DMSO has stronger polarity and water miscibility than GF, resulting in faster water diffusion and promoting solvent removal [51]. The viscosity also affected the gel formation of ISG since the viscosity of ZG was greater than that of ZD, contributing to a reduction in water diffusion into the system. Therefore, the rapid phase inversion of ZD from the solution into a gel-like state was evident in the low viscosity formula because of the ease of solvent exchange [31]. However, all ZD ISG showed rapid and complete transformation within 30 min. Rapid transformation while injecting makes periodontitis treatment more practical, improves patient compliance, and avoids formula leaks from the periodontal pocket [52].

**Figure 4:**
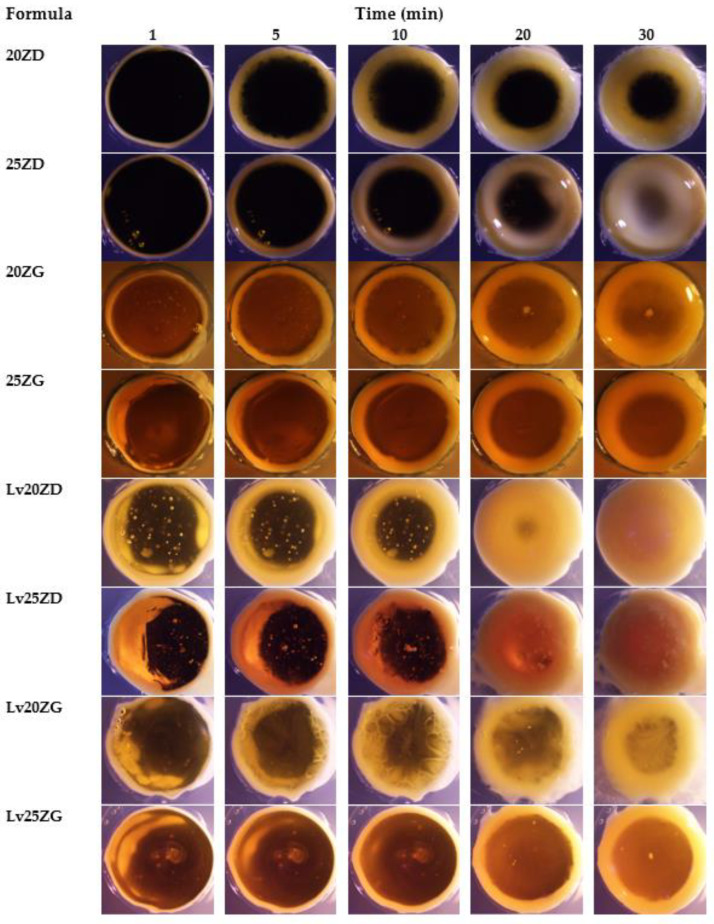
Gel formation of drug-free and Lv-loaded zein-based after contact with agarose gel with different time intervals under a stereomicroscope at a magnification of ×12.

Moreover, the transformation of these ISGs in agarose agar was investigated for a cross-section view of gel formation as a generation of a thinner opaque layer at the time of the transformed region at the interface of the contact surface (Figure 4). The phase transformation of zein-based ISG developed the formation of the outer layer gel at the initial stage, and the polymer interior slowly converted into the stable matrix. All formulations initially presented the formation of a gel layer at 1 min after contact with the aqueous phase of agarose gel. Lv20ZD and Lv25ZD showed the full opaque gels in the agarose well at 25 min. Being less viscous, the DMSO more efficiently provoked gel formation of the zein-based ISG than GF because the rate of solvent exchange was the key parameter for gel formation [26,27]. The trend of viscosity of the solvents was glycerol formal > triacetin > 2-pyrrolidone > isopropyl myristate > *N*-methyl pyrrolidone > DMSO [53]. Moreover, rapid gel formation was observed for the ISG systems dissolved in DMSO because the rather high polarity of this solvent could rapidly diffuse into water and promote solvent removal [37,54]. By comparison, the obtained dense gel of drug-free and Lv-loaded 25% zein ISGs seemed to act as a barrier retarding the solvent exchange and thus hamper a fully opaque gel formation. The looser zein gel-like matrices of drug-free and Lv-loaded 25% zein ISGs were nearly fully filled in the agarose well, as shown in Figure 4. The zein gel formation was promoted with the addition of Lv. Therefore, the dissolved Lv enhanced the gel formation of the exchanging area from its hydrophilicity. Five percent of the doxycycline hyclate ISG transformed into a matrix using the 30% *w*/*w* Eudragit RS matrix-forming agent for periodontitis treatment [29], whereas, 0.75% vancomycin HCl promoted gel formation of saturated fatty acid-based ISG for patients with joint infection after total knee arthroplasty [50]. The high transformation ability indicated the high aqueous permeability of forming matrices. A tiny volume of less than 50 µL of antibacterial agent-loaded ISG was practically injected into the periodontal pocket for periodontitis treatment [25,52]. Therefore, the crevicular fluid could contact this large surface area per mass of ISG and induce a phase transformation into the zein matrix for modulating drug release.

### 3.4. Drug Content and In Vitro Drug Release Study

**Figure 5:**
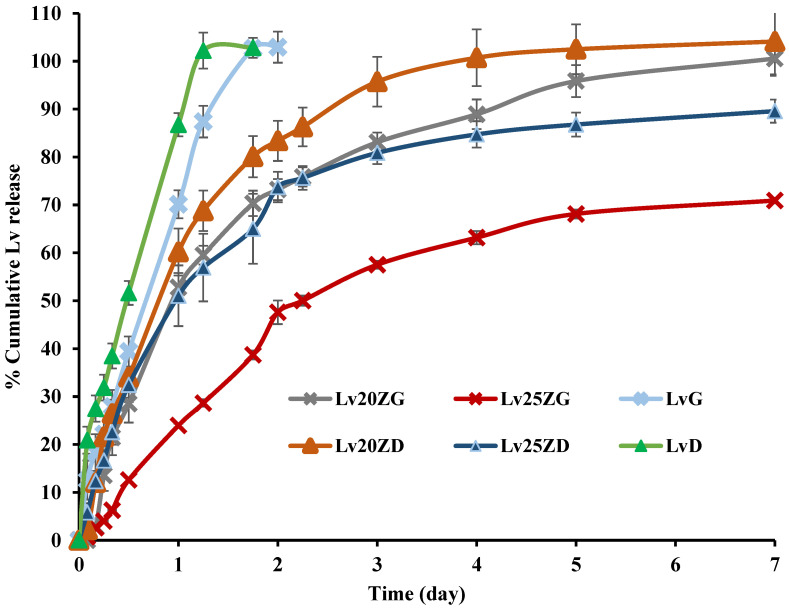
Release of Lv from zein-based ISGs formulations using the cup method (*n* = 3).

The Lv content amount in the LvD, LvG, Lv20ZD, Lv25ZD, Lv20ZG, and Lv25ZG was 100.08 ± 0.74, 97.41 ± 0.16, 102.62.05 ± 0.82, 101.46 ± 0.88, 98.46 ± 0.82, and 99.11 ± 1.06, respectively. These formulations were prepared by a simple mixing process; thus, their amounts of Lv content were close to 100%. The drug-loaded zein ISGs were tested for their in vitro cumulative drug release using the cup method to mimic the surroundings of a periodontal pocket [28,29]. Various investigators have employed the cup method to undertake the drug release behavior of in situ forming drug delivery systems. Using this technique, doxycycline hyclate is released sustainably from ethyl cellulose, beta-cyclodextrin, and poly(D,L-lactide-co-glycolide ISGs [55,56,57]. The control groups, such as LvD and LvG, released the drug completely at 28 h and 36 h, respectively, whereas the zein-based ISGs gradually released the Lv for 7 days into PBS pH 6.8 solution (Figure 5). The higher concentration zein loading resulted in more prolonged LV release in which the use of GF more retarded the drug liberation from ISG. The denser matrices from high-loading polymers more efficiently sustained ISG drug release owing to their effective barrier effect against solvent removal and drug diffusion [55]. In addition, the less viscous DMSO series ISG facilitated the water entry and faster drug release into the medium than the GF series ISG. The use of GF as a solvent and 25% zein encouraged the prominent retardation of Lv release, as shown in Figure 5, which confirmed the efficacy of this protein as the matrix-forming agent of ISG to control the drug release. The commercial Atridox^®^ using doxycycline hyclate as an antimicrobial agent loading into 33.03% poly(D,L-lactide) solution sustained the drug release for 7 days [58,59]. The periodontal pocket of periodontitis is typically cleaned by dentists with an irrigating solution and flushed with an irrigating solution such as chlorhexidine mouthwash to diminish plaque bacteria before administration with the sustained antibacterial dosage form [60]. Hence, this study’s Lv-loaded zein-based ISGs formulation could control drug release for 7 days. Thence, zein-based ISGs efficiently exhibited controlled Lv liberation with local prolonged drug release in this pocket area for promoting patient compliance owing to less frequency of drug administration [61].

For non-swellable cylindrical matrices, the values of *n* from the Korsmeyer–Peppas equation are 0.45 and 1.0 for Fickian and case-II transport, respectively [62]. When the value of *n* is >0.45 and <1.0, the release was claimed as non-Fickian [62,63]. A value of *n* = 1 indicates a zero-order release [63,64]. The regression coefficient (r^2^) value and diffusion exponent value (*n*) obtained from the drug release profile of Lv-loaded zein-based ISGs fitting to different mathematical equations are shown in Table 3. The best model that mostly complied with release profiles was the first-order kinetic indicating that drug release was a fraction of the remaining drug in the matrix. Therefore, Lv release is mainly driven by diffusion of the medium into the zein composite matrix. Nonetheless, the drug release from other matrix-forming agent-based ISGs fabricated from polymers or fatty acids mostly fitted well with Higuchi’s equation with Fickian diffusion drug release [50]. However, doxycycline hyclate released from 5–15% *w*/*w* ethyl cellulose, 15–25% *w*/*w* bleached shellac, and 15–30% *w*/*w* Eudragit RS formula using the cup method fitted well with first order model [29]. Metronidazole-loaded polycaprolactone/alginic acid-based polymeric film in treating periodontal diseases showed a burst drug release followed by a gradual release. It fitted well with the first-order kinetic model indicating that drug release is a fraction of the drug remaining in the matrix [65]. When liquid diffusion and polymer relaxation rates are of the same magnitude during immersion in the release medium, anomalous or non-Fickian diffusion is dominant [66]. As the rate of liquid diffusion is slower than the relaxation rate of the polymeric chains, the diffusion is Fickian. Meanwhile, the case II transport is dominant when the relaxation process is very slow compared with the diffusion [66]. Normally, hydrophilic drugs liberate much more rapidly than hydrophobic drugs, particularly at the initial time with burst release while the ISG is still solidifying [67]. Nevertheless, gradual drug release was achieved from these zein-based ISGs without burst release. The viscous zein gel formation over time before completely transforming into zein matrices successfully retarded an initial drug diffusion.

### 3.5. Scanning Electron Microscopy (SEM)

**Figure 6:**
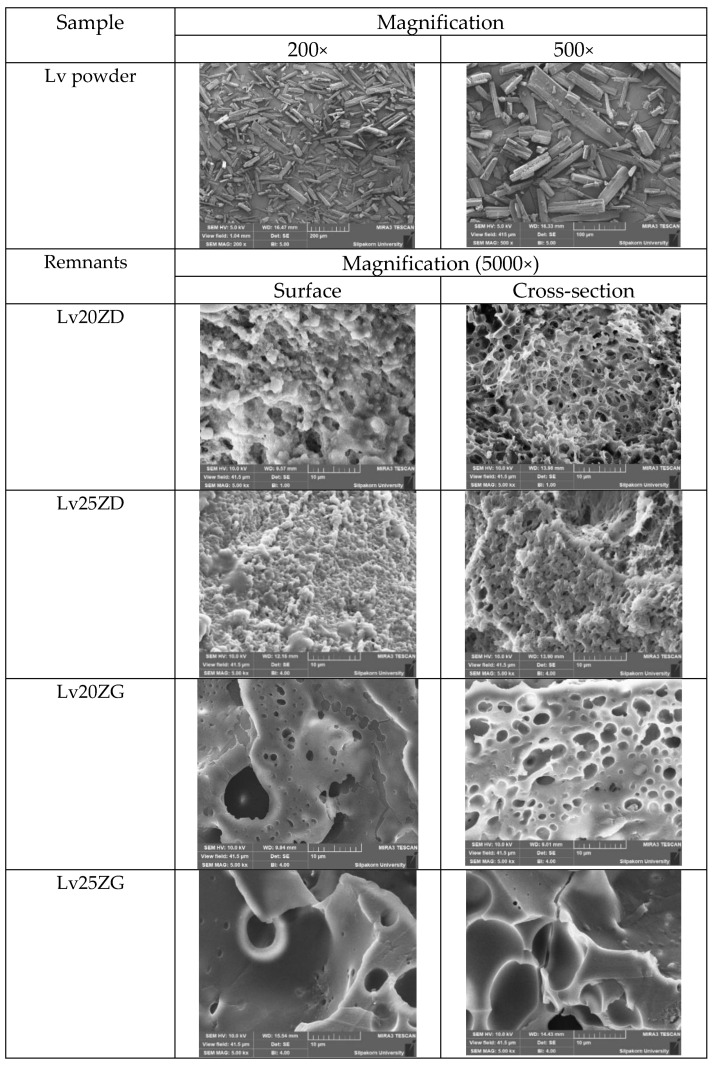
SEM images of intact Lv powder at different magnifications and freeze-dried Lv-loaded zein-based ISGs at a magnification of 5000×.

The SEM photographs showed the surface and cross-section topographies of Lv-loaded 20% and 25% zein-based ISG remnants after drug release (Figure 6). The crystalline form with various sizes of intact Lv powder was evident. The concentration of zein markedly influenced surface and inner topographies, such as the interconnected porous structure of ISG. Lv20ZD presented a notably more porous scaffold structure that corresponded with it releasing more drug as previously described when compared to the Lv20ZD. When the Lv20ZD solutions were injected into the buffer release medium solution, rapid diffusion of a less viscous formulation of DMSO progressed into an aqueous phase via solvent exchange leading to the simultaneous emergence of a porous structure with a sponge-like topography and a porous surface [27]. The pore sizes of the structure decreased when the amount of zein increased. This characteristic corresponded with the in vitro gel formation in which the obtained gels were much more solid and opaque as the zein concentration was increased (Figure 3). These pores performed as main channels for releasing the Lv molecules from the inner zein gel or matrix into the release medium. The dense matrix topography, both on the surface and cross-section views, was evident apparently for Lv25ZD to more efficiently retard the drug release. The greater agglomeration of zein fibrous was seen in the higher loaded zein formulation (Figure 6) since zein typically performs as a globular protein in nonaqueous solutions and has the conformational characteristics of more conventionally behaving globular proteins [41]. Additionally, the higher viscous ISG absorbs less water, causing a slow-moving solvent diffusion and accomplishing a smooth surface [44,68]. The role of the polymer concentration in modulating drug release has been previously reported from other ISGs for periodontitis treatment [29,55] and corresponded with this investigation.

However, the SEM image of two ISGs employing GF as a solvent demonstrated a significantly different morphology. They did not present an interconnected porous structure, as presented in Figure 6. No remaining Lv crystals appeared in test remnants, suggesting that the drug release was nearly complete; nevertheless, some crystals presented at the inner part of the zein matrix of Lv25ZG. Both formulations showed a combination of dense and porous structures. The transformed zein matrices were thicker and denser than the previously mentioned ISGs. The less porous matrix of Lv25ZG promoted its ability to release the drug more slowly than the Lv20ZG matrix. Typically, the pore formation of the ISG is related to the solvent exchange rate. Including a greater zein concentration or using a viscous solvent caused the gels to resist entering the water and the solvent leaching out, resulting in a less interconnected porous structure [69]. Therefore, this characteristic emphasized the retardation of drug release from the zein ISG. These remnant microphotographs after the release test revealed that the pores had been formed in the matrix and indicated that the involvement of solvent exchange was responsible for modulating the drug release from the zein ISG matrices.

### 3.6. X-ray Computed Microtomography (μCT)

**Figure 7:**
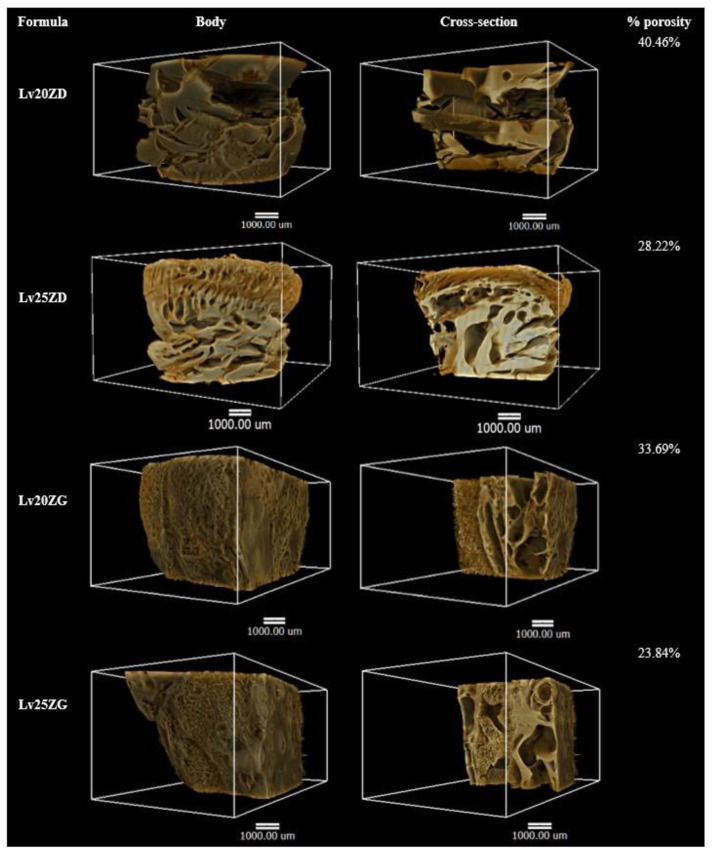
X-ray tomography image and % porosity using X-ray tomography of the freeze-dried Lv-loaded zein-based ISGs.

X-ray tomographic imaging is a nondestructive technique for visualizing interior features within solid objects and obtaining digital information on their 3D geometries and properties, which can be acquired using synchrotron sources, including the different possible contrast modes that can be exploited [70]. X-ray tomographic images of the Lv-loaded zein-based ISG remnants obtained from a synchrotron light source and their 3D volume and the cross-section with the pores inside are depicted in Figure 7. Their skin-like feature is clearly shown on the Lv25ZD remnant, resulting in more retardation of drug release than Lv20ZD. In addition, the Lv25ZD’s % of porosity was notably less than the Lv20ZD. Moreover, the analysis in 3D indicated that both Lv20ZD and Lv25ZD have more % porosity than Lv20ZG and Lv25ZG, respectively. This suggested that GF solvent might have some negative effect on porosity relative to DMSO. However, it was found that both Lv25ZD and Lv25ZG have significantly decreased porosity. This indicated that incorporating more zein ISG could help develop a denser matrix that prolonged drug release, as described previously. The result of X-ray tomographic imaging provides the support evident to topography obtained from SEM and drug release behavior. In addition, the lower porosity value of the more highly incorporated zein ISG indicated its denser matrix formation and prolonged drug release. Therefore, the X-ray tomographical zein remnant images could support the explanation for topography from SEM and drug release behavior. Furthermore, the porosity and pore-connectivity ensured the solvent removal and drug diffusion from in situ gel systems formed into scaffolds. Numerous studies presented that the injectable biomaterial dosage forms formed scaffolds in situ and were useful as localized or systemic drug delivery systems [26,27,68,71]. X-ray tomographic imaging is, therefore, a useful technique for scrutinizing 3D geometries and porosity characteristics of the ISG systems.

### 3.7. Antimicrobial Activities

**Figure 8:**
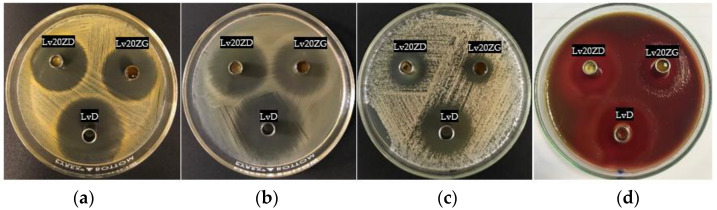
Photographs of the inhibition zone of Lv20ZD (upper-left corner cup) and Lv20ZG (upper-right corner cup) ISG formulations against *S. aureus* (**a**), *E. coli* (**b**), *C. albicans* (**c**), and *P. gingivalis* (**d**) and the control group (LvD) at the bottom cup.

The inhibition zone of solvents, drug-free and Lv-loaded zein ISGs against *Staphylococcus aureus* (ATCC 6538), *Escherichia coli* ATCC 8739, *Candida albicans* ATCC 10,231 and *Porphyromonas gingivalis* ATCC 33,277 from the antimicrobial test is shown in Figure 8 and their inhibition diameters are shown in Table 4. These microbe species are associated with periodontitis disease, especially pathogens such as *Porphyromonas gingivalis* ATCC 33,277. DMSO and GF are the organic vehicles being utilized as solvents in depot dosage forms by virtue of their safety with low toxicity. DMSO is employed as a vehicle in formulating an injectable subcutaneous implant [72,73]. LD50 for intravenous and subcutaneous injections of DMSO in rats is 5.3 and 12 g/kg, respectively [74]. In addition, it can be used as a vehicle to dissolve the polymer of in situ-forming implants [75]. GF has been known as a parenteral solvent to dissolve a wide range of aqueous insoluble compounds [53,76]. It was used as a co-solvent for veterinary formulations to obtain the high viscous systems to control drug release, and it has been used as the injectable solvent of the in situ-forming implant system [21,76]. In addition, GF was also used as the solvent for the ivermectin-loaded subcutaneous injection in young pigs [77], and for the pyrethroid insecticide, deltamethrin-injected suspension exhibited a prolonged half-life and a slower rate of clearance [78]. The LD_100_ of GF by intravenous injection in mice is 3 g/kg [53,79]. While the safety data with various medical applications of DMSO, GF, and zein are available, the developed formulations need to be further investigated in a clinical experiment as to their safety. From this study, the antimicrobial activities of GF were more potent than DMSO, especially against *C. albicans* though not against *P. gingivalis,* as shown in Table 4. Although the viscosity of GF is higher than that of DMSO, it could inhibit microbial growth more efficiently. Organic solvents such as DMSO and GF have a potential solubilizing effect against lipids in the cell walls of microbes; therefore, they could inhibit microbial growth. The Lv-free zein-based ISG showed a smaller inhibition zone diameter than its solvent, especially 25ZD, on account of the fact that the apparent viscous characteristic owing to the addition of zein could impede the outward diffusion of solvent from these systems into inoculated media. The Lv solutions as the control group significantly (*p* < 0.05) exhibited large inhibition zone diameters due to the drug freely diffusing, excluding *C. albicans*. Their inhibition against *C. albicans* seemed to be caused by the solvent, especially from GF, and not from the drug compound since their clear zones were less than those of the solvents. However, The Lv-zein-based ISG showed a significantly (*p* < 0.05) smaller inhibition zone diameter for *S. aureus*, *E. coli,* and *P. gingivalis* than the Lv solutions as the control group. Incorporating zein retarded the drug diffusion; therefore, the smaller inhibition clear zone was evident with the dependent zein concentration. This result corresponded with the drug release behavior of Lv from prepared ISGs as previously described. In addition, Lv25ZD did not show the inhibition zone against *C. albicans*. The lesser amount of DMSO and viscous formulation hampered this antifungal activity. A smaller clear zone was obtained with higher zein loading. Thus, the zein matrices, after phase transformation retarded outward drug diffusion with prolongation of drug release and diminished the inhibition zone diameter. These similar results have been noted formerly for the matrices that transformed from ISG prepared by employing poly(D,L-lactide-co-glycolide) [57], natural resins [27,28], polymers [29,55], and saturated fatty acids [30,50] as matrix-forming agents of in situ-forming systems. For periodontitis, the precise accountability that such microbe types and species play, singularly or in combination, in the pathogenesis stage of periodontal breakdown remains to be determined. Unlike general major infections, all the suspected periodontal pathogens are indigenous to the oral flora, and *C. albicans* causes refractory periodontitis [80]. *S. aureus* could be isolated from the periodontal pockets of patients with aggressive periodontitis, and E. coli was considered a microorganism common in patients with periodontitis [81]. Therefore, the effective ISG for inhibition of all related pathogens of periodontitis in a controlled drug-release manner emerged as an attractive localized dosage form. Practically, the medicated in situ forming gel showed superior clinical outcomes compared to scaling and root planing alone, which could be associated with the presence of GF that is not only competent in periodontitis treatment but also acts as a good vehicle of ISG to deliver the drug into the periodontal pocket. Atridox^®^, Atrisorb-D^®^, and FreeFlow™ are commercial products prepared as ISGs for periodontitis treatment; nevertheless, these products are composed of rather expensive matrix-forming agents. Therefore, searching for new material, such as zein as a matrix-forming agent, is beneficial for ISG development. A commercial product such as Atridox^®^ is a subgingival controlled-release product composed of two syringe mixing systems. The first syringe contains a bioabsorbable, flowable polymeric formulation composed of poly(DL-lactide) dissolved in organic solvent. After mixing with the first syringe, the second pre-filled syringe contains 44 mg doxycycline hyclate powder or 8.8% *w*/*w* doxycycline hyclate. The preparation of pre-filled syringes enables us to enhance drug stability. In addition, Lv-incorporated zein-based solvent removal phase inversion ISG fabricated without an aqueous phase as a component and no heating could prevent drug degradation.

Lv is a broad-spectrum, third-generation fluoroquinolone antibiotic that diffuses through the bacterial cell wall and acts by inhibiting DNA gyrase and topoisomerase IV, an enzyme required for DNA replication [31,32]. The antimicrobial activities of Lv were noted with MIC (minimum inhibitory concentration) against *S. aureus, E. coli,* and *P. gingivalis* of 0.12 μg/mL [82], ≤0.12 μg/mL [83] and 8 μg/mL [84], respectively. The concentration of whole drug release from this study was 43.75 μg/mL (100% release); therefore, the 18.29% drug release on or around 6 h, all formulations except Lv25ZG could achieve the MIC of Lv against *P. gingivalis*. From these results, Lv20ZG and Lv20ZD exhibited a controlled drug release for 7 days with efficient antimicrobial activities against all test microbes; nevertheless, the X-ray imaging indicated that the transformed Lv20ZD matrix had an incomplete inner structure. Thus, Lv20ZG presented as the more potential ISG formulation for periodontitis treatment. Moreover, this ISG released the drug above the MIC against pathogens such as *P. gingivalis*. Practically, the local drug delivery is designed to localize high-dose drugs at target size for effective treatment. The release of a hydrophilic drug such as 1% levofloxacin HCl from zein-based solvent removal phase inversion ISG was sufficient and above the MIC against microbes as mentioned above because the inoculated agar media composed of lower amounts of water than that in the release medium. Therefore, its concentration after diffusion in a controlled-release manner into an agar medium could efficiently inhibit the antimicrobial activities, as shown in Table 4.

## 4. Conclusions

The solvent removal phase inversion zein-based ISG formulation was successfully prepared using DMSO and GF as the solvents. The viscosity and force-work of injection of zein ISG prepared using GF were notably higher than those using DMSO, whereas the gel formation was slower. The higher zein loading showed a more prolonged Lv release. The denser matrix from ISG prepared with GF as a solvent exhibited more prolonged drug release. SEM and X-ray imaging revealed the different topographies and 3D structures of dried ISG. Their % porosity value correlated well with their phase transformation and drug release behavior. The addition of zein retarded the drug diffusion; therefore, the inhibition clear zone was smaller and corresponded with the drug release. The concentration of drug release from all formulations could be attained above MIC against pathogen microbes and exhibited a controlled drug release for 7 days. Lv20ZG exhibited appropriate viscosity, Newtonian flow, acceptable gel formation, and injectability, and prolonged Lv release for 7 days with efficient antimicrobial activities against *S. aureus, E. coli*, *C. albicans,* and *P. gingivalis*; thus, it is the potential ISG formulation for periodontitis treatment. While safety data is available with various medical applications of zein and GF, further clinical experiment for the safety of this developed ISG formulation is essential.

## Figures and Tables

**Table 1:** Composition of Lv-loaded zein-based ISG formulations.

Formula	Levofloxacin HCl (Lv)	Zein	DMSO	Glycerol Formal (GF)
	(% *w*/*w*)	(% *w*/*w*)	(% *w*/*w*)	(% *w*/*w*)
20ZD	-	20	80	-
25ZD	-	25	75	-
20ZG	-	20	-	80
25ZG	-	25	-	75
Lv20ZD	1	20	79	-
Lv25ZD	1	25	74	-
Lv20ZG	1	20	-	79
Lv25ZG	1	25	-	74
LvG	1	-	-	99
LvD	1	-	99	-

**Table 2:** Injectability properties of zein-loaded ISG formulations (*n* = 3).

Formula	Injectability Force (N)	Work of Injectability (N.mm)
20ZD	1.02 ± 0.08	7.51 ± 0.15 ^c^
25ZD	1.19 ± 0.05	9.13 ± 0.21 ^c,e^
20ZG	1.07 ± 0.10 ^a^	13.75 ± 0.95 ^f^
25ZG	3.02 ± 0.04 ^a^	42.31 ± 1.23 ^f^
Lv20ZD	1.00 ± 0.04	7.09 ± 0.17 ^d^
Lv25ZD	1.14 ± 0.03	12.34 ± 0.21 ^d,e^
Lv20ZG	1.15 ± 0.06 ^b^	15.20 ± 0.40 ^g^
Lv25ZG	3.07 ± 0.13 ^b^	44.75 ± 1.14 ^g^

The superscripts (a–g) in the column represent a significant difference within the tested formulations (*p* < 0.05).

**Table 3:** The regression coefficient (r^2^) value and diffusion exponent value (*n*) obtained from the drug release profile of Lv-loaded zein-based ISGs fitting to different mathematical equations.

Formula	Zero-Order	First-Order	Higuchi’s	Korsmeyer–Peppas		
	r^2^	r^2^	r^2^	r^2^	*n*	Release Mechanism
LvD	0.9437	0.9693	0.9730	0.9809	0.599 ± 0.055	non-Fickian diffusion
LvG	0.9712	0.9336	0.9175	0.9774	0.797 ± 0.054	non-Fickian diffusion
Lv20ZD	0.8449	0.9930	0.9578	0.9597	0.530 ± 0.015	non-Fickian diffusion
Lv25ZD	0.7644	0.9774	0.9228	0.9542	0.387 ± 0.020	non-Fickian diffusion
Lv20ZG	0.8345	0.9855	0.9345	0.9451	0.574 ± 0.045	non-Fickian diffusion
Lv25ZG	0.7944	0.9622	0.9408	0.9418	0.477 ± 0.010	non-Fickian diffusion

**Table 4:** Inhibition zone diameters of DMSO, GF, and Lv-loaded zein-based ISG formulations against *S. aureus*, *E.* coli, *C. albicans,* and *P. gingivalis* (*n* = 3).

Formula	Inhibition Zone + S.D. (mm)			
	*S. aureus* ATCC 6538	*E. coli* ATCC 8739	*C. albicans* ATCC 10231	*P. gingivalis* ATCC 33277
DMSO	12.3 ± 0.5 ^a^	16.0 ± 0.8 ^a^	25.7 ± 1.2 ^a^	17.3 ± 0.5 ^a^
GF	15.7 ± 1.2 ^c^	16.3 ± 0.5 ^c^	31.3 ± 1.2 ^c^	13.3 ± 0.5 ^c^
20ZD	11.3 ± 0.5	14.0 ± 0.0	13.7 ± 2.1	16.0 ± 0.8
25ZD	-	-	10.3 ± 0.5 ^a^	-
20ZG	12.7 ± 0.9 ^c^	15.0 ± 0.8	14.0 ± 1.4 ^c^	14.0 ± 0.8
25ZG	-	-	14.7 ± 0.9 ^c^	-
LvD	37.7 ± 0.5 ^a,b^	38.3 ± 1.2 ^a,b^	19.7 ± 1.7 ^a^	39.7 ± 1.7 ^a,b^
LvG	37.0 ± 0.8 ^c, d^	35.7 ± 0.5 ^c, d^	27.3 ± 0.9 ^d^	33.7 ± 0.5 ^c,d^
Lv20ZD	34.3 ± 0.5 ^b^	32.0 ± 0.8 ^b^	16.7 ± 2.1	35.3 ± 1.2 ^b^
Lv25ZD	34.3 ± 0.5 ^b^	31.3 ± 0.5 ^b^	-	29.0 ± 0.8 ^b^
Lv20ZG	34.3 ± 0.5 ^d^	30.7 ± 0.5 ^d^	17.0 ± 1.4 ^d^	29.3 ± 1.7 ^d^
Lv25ZG	32.3 ± 0.5 ^d^	29.7 ± 0.5 ^d^	12.0 ± 1.6 ^d^	26.0 ± 0.8 ^d^

“-“ = No inhibition zone; the superscripts a–d indicate a significant difference (*p* < 0.05) using one-way ANOVA followed by an LSD post-hoc test.

## Data Availability

The data presented in this study are available on request from the corresponding author.
